# The Effect of an After-School Physical Activity Program on Children’s Cognitive, Social, and Emotional Health during the COVID-19 Pandemic in Nova Scotia

**DOI:** 10.3390/ijerph19042401

**Published:** 2022-02-19

**Authors:** Hilary A. T. Caldwell, Matthew B. Miller, Constance Tweedie, Jeffery B. L. Zahavich, Ella Cockett, Laurene Rehman

**Affiliations:** 1 School of Health and Human Performance, Dalhousie University, Halifax, NS B3H 4R2, Canada; hilary.caldwell@dal.ca (H.A.T.C.); matthew.miller@acadiau.ca (M.B.M.); ctweedie@dal.ca (C.T.); zahavich@dal.ca (J.B.L.Z.); ecockett@dal.ca (E.C.); 2 Healthy Populations Institute, Dalhousie University, Halifax, NS B3H 3E2, Canada; 3 School of Kinesiology, Acadia University, Wolfville, NS B4P 2R6, Canada

**Keywords:** physical activity, PROMIS, children, COVID-19 pandemic, public health

## Abstract

Children’s physical activity participation declined during the COVID-19 pandemic, and these negative changes could lead to longer-term impacts on children’s cognitive, social, and emotional health. Purpose: To determine parent/caregivers’ perceptions of their children’s cognitive function, peer and family relationships, life satisfaction, physical activity, sleep, positive affect, and global health, before and after participating in the Build Our Kids’ Success (BOKS) programming at after-school programs in Fall 2020. Methods: Parents of children participating in the BOKS programming at after-school programs in Nova Scotia, Canada, were recruited. At baseline, 159 parents completed the National Institutes of Health (NIH) Patient-Reported Outcomes Measures Information System (PROMIS) parent-proxy questionnaire, and 75 parents completed the measures at follow-up. Independent t-tests were used to determine if there were differences between baseline and follow-up Parent Proxy Questionnaire data. Results: All NIH PROMIS outcome variables at baseline and follow-up were within normal limits (Adjusted T-Scores: 46.67 ± 7.15 to 50.04 ± 7.13). There were no significant differences in life satisfaction (t(188) = −1.05, *p* = 0.30), family relationships (t(189) = 0.31, *p* = 0.76), cognitive function (t(199) = −1.16, *p* = 0.25), peer relationships (t(192) = −1.86, *p* = 0.06), positive affect (t(195) = 0.25, *p* = 0.81), global health (t(216) = −0.43, *p* = 0.67), physical activity (t(202) = 0.787, *p* = 0.732), sleep disturbance (t(193) = 1.72, *p* = 0.087), or psychological stress (t(196) = 1.896, *p* = 0.059), from baseline to follow-up. Conclusions: Parent-proxy questionnaires suggested that the BOKS programming had a protective effect on children’s health behaviours and cognitive, social, and emotional health as values remained within normal limits and were not impacted by the public health restrictions during the second wave of the COVID-19 pandemic in Nova Scotia.

## 1. Introduction

The COVID-19 pandemic, and its associated public health restrictions, greatly impacted the wellbeing and mental health of children and youth as their daily lives were disrupted by school closures, limited social interactions, and the cancellation of in-person extra-curricular activities, sports, and recreation opportunities. Canadian youth reported that they missed being able to leave the house, go to school, and see their friends during the pandemic [1]. Families experienced hardships during the pandemic, including job loss, income loss, caregiving burdens, and illness, and a higher number of hardships were associated with lower parental and child wellbeing [2]. Parents in the United States also reported worsening mental and behavioral health for themselves and their children [3]. A study from Nova Scotia, Canada, reported that youth felt bored, relaxed, depressed, safe, and worried during the pandemic and most youth participants felt they were missing important life events and seeing their friends at school [4]. It remains unclear how to best intervene to support children and youth’s health and wellbeing during the pandemic; however, interventions and activities are needed to ensure long-term consequences are mitigated.

Physical activity participation is associated with multiple physical, mental, and social health benefits in children and youth, and higher fitness or physical activity participation is associated with better cognitive and improved mental health in children and adolescents [5,6]. During the first and second waves of the COVID-19 pandemic, less than 5% of Canadian children and youth met the recommended 24-Hour Movement Guidelines for Children and Youth, and parents reported that children engaged in less physical activity and more screen time than before the pandemic [7,8]. Public health restrictions across Canada limited access to spaces and places for physical activity, such as parks and recreation facilities, and children and youth spent less time outdoors than before the pandemic [9]. During the first wave of the pandemic (Spring 2020), children and youth living in the province of Ontario, a region with a high prevalence of COVID-19, experienced the greatest declines in outdoor play and time spent outdoors [9]. At this time, provincial and national parks were closed, schools were closed to in-person learning, and sports practices and group recreation opportunities were not permitted [9].

In early Fall 2020, while most of Canada experienced a severe second wave of COVID-19, case counts remained low in Atlantic Canada (New Brunswick, Nova Scotia, Prince Edward Island, Newfoundland). At this time, children and youth living in Atlantic Canada participated in more moderate-to-vigorous physical activity (MVPA) and experienced smaller declines in outdoor play and time spent outside compared to those living in the province Quebec, the region with the highest COVID-19 prevalence at the time [10]. These differences may be attributable to the less severe public health restrictions in Atlantic Canada versus Quebec. For example, organized sports were permitted in Atlantic Canada, but not in parts of Quebec or Ontario [10]. The Build Our Kids’ Success Program (BOKS) was implemented in after-school programs in Halifax, Nova Scotia, Canada, in Fall 2020. During its delivery, Nova Scotia entered its second wave of COVID-19, and further restrictions were implemented province-wide [11]. While schools and childcare centres remained open to in-person learning at this time (including before- and after-school childcare programs), sports and recreation facilities were closed, and gathering limits were re-instated [11]. With sport and recreation facilities closed and programming cancelled, children and youth’s physical activity opportunities outside of school declined, while physical activity opportunities at school and childcare continued.

School settings provide opportunities for children to be active across the school day, including to and from school and before and after school programs [12]. Before- and after-school programs are a useful way to supplement physical activity time for children and youth, and they have been effective at improving children and youth’s physical activity levels and preventing obesity [13]. This study assessed the effectiveness of the BOKS program applied in after-school settings. BOKS has a mission to ‘make physical activity and play a part of every child’s day’ and consists of a variety of free, fun, and engaging resources to support children to develop a lifelong commitment to health and wellbeing. BOKS programming resources include full physical activity plans, short movement bursts and movement-based games, activities, and resources designed for school or home use [14]. Several studies have examined the effectiveness and impact of BOKS applied in before-school settings. One study reported that students who participated in BOKS accumulated more steps and physical activity on BOKS days compared to days without BOKS programming [15]. Improvements in body mass index, percent body fat, aerobic performance, and student engagement were also demonstrated among students enrolled in BOKS compared to students not enrolled in the program [16,17]. To be most effective, after-school programs should be implemented in schools rather than community settings and offered at least two days per week [13]. It is still unclear if participation in the BOKS program has favourable impacts on students’ physical activity enjoyment, physical literacy, cognitive function, or wellbeing. 

The purpose of this study was to determine parents’ perceptions of cognitive function, peer and family relationships, life satisfaction, physical activity, affect, sleep, and global health among their children who were enrolled in an after-school childcare program that offered BOKS programming in Nova Scotia, Canada, from October to December 2020. 

## 2. Materials and Methods

This study was approved by the Dalhousie University Research Ethics Board (REB# 2019-5024). At baseline, we recruited 159 parents of children participating in the BOKS program at elementary schools in the Halifax Regional Centre for Education in Halifax, Nova Scotia, Canada. At follow-up, 79 parents completed surveys. The BOKS program consists of free, fun, and engaging resources to meet the needs of Canadian kids from early childhood through high school, to become active and develop a lifelong commitment to health and fitness. BOKS program resources include full-length physical activity plans (20–45 min), short movement breaks (1–10 min), and movement-based games, activities, and resources designed for either school or at-home use. The full-length physical activity plans include a warm-up activity (i.e., adventure run, BOKS Says), running-related activity (e.g., running relays, musical run), skill of the week (i.e., planks, sprints), game (i.e., crab walk soccer, red light-green light), cool down (i.e., deep breaths, full-body stretch), and a BOKS Bits nutrition talk. The short movement breaks are designed to keep children active throughout activity and may include activities such as an ABC Workout, Bingo Burst, or BOKS Says. More detail about BOKS programming and activities is available for free from BOKS Canada [18]. On average, BOKS programming was implemented for 70 min per week. 

### 2.1. Procedures

Parents of students of all genders in grades 3, 4, 5, and 6 who were enrolled in the Excel after-school program were recruited to participate in this study. At baseline in October 2020, 159 participants completed the National Institutes of Health (NIH) Patient-Reported Outcomes Measures Information System (PROMIS) parent-proxy questionnaire online using Dalhousie University’s Opinio software program. At follow-up in December 2020, 79 parents participated in the study. Six subscales (life satisfaction, family relationships, cognitive function, peer relationships, positive affect, and global health) of the NIH PROMIS parent proxy report questionnaires were included [19,20,21,22,23]. Parents responded to four-item scales for psychological stress and sleep, seven-item scales for cognitive function, peer relationships, and global health, eight-item scales for family relationships, positive affect, and life satisfaction. For example, questions included: “how often does your child have fun with friends?”, “Your child has trouble remembering to do things such as school projects or chores”, and “My child felt stressed”. Each question had five response options, such as excellent to poor, always to never, or not at all to very much. The physical activity was seven-items long and asked how frequently children were active in several contexts during the week, such as “how many days did your child exercise really hard for 10 min or more?” and “how many days did your child run for 10 min or more?”. The five response options for physical activity questions were 0 days, 1 day, 2–3 days, 4–5 days, or 6–7 days. Each response option to each question was given a score of 1–5, and then scores within each scale were summed (raw scores). PROMIS measures have a common metric, the T-Score, with a mean of 50 and a standard deviation of 10 where 50 equals the means for the U.S. general population [24]. Raw scores were converted into adjusted T-scores, and both raw and T-scores were reported. 

### 2.2. Analysis

Data were analyzed in SPSS Version 26. Means and standard deviations for raw and adjusted T-scores for all NIH PROMIS outcomes were calculated. Chi-squared tests were used to determine differences in the proportions of children’s grades and gender at baseline and follow-up. Given the difference in sample sizes between pre- and post-intervention data, it was not appropriate to use paired data analyses methods. Independent t-tests were used to determine if there were significant differences between pre and post parent-proxy questionnaire data. Significance was set at *p* ≤ 0.05.

## 3. Results

Participant characteristics are included in Table 1. At baseline and follow-up, most children were in grade 4 (60–65%), with smaller proportions in grade 5 (30%) and 6 (5–6%). The proportions of children’s grades (𝝌^2^ = 2.275, *p* = 0.517) were not different between the two samples. Gender was similarly distributed between males and females (𝝌^2^ = 1.762, *p* = 0.414). All NIH PROMIS outcomes variables at baseline and follow-up were within normal limits (adjusted T-Scores: 46.67 ± 7.15 to 50.04 ± 7.13). There were no significant differences in raw or adjusted T-scores for life satisfaction, family relationships, cognitive function, peer relationships, positive affect, global health, physical activity, sleep disturbances, or psychological stress between baseline and follow-up (*p* > 0.05, see Table 2). 

## 4. Discussion

This study assessed parents and guardians’ perceptions of their children’s cognitive, social, and emotional health among children who participated in the BOKS after-school physical activity program in Nova Scotia, Canada, from October to December 2020. Despite the second wave of the COVID-19 pandemic and related public health restrictions in Nova Scotia, parents reported values within the normal range for all cognitive, social, and emotional health indicators, and there were no changes between baseline and follow-up. The results suggest that participation in the BOKS after-school program may have had a protective effect on children’s health behaviours and cognitive, social, and emotional health. 

Children’s movement behaviours, including physical activity, sedentary behaviour, and sleep, were greatly impacted by the COVID-19 pandemic, as reported in previous research. For example, in a study of movement behaviours in 1500 children and youth from across Canada in Fall 2020, the same timepoint as our study, 59% of children and youth met the sleep recommendations for their age and only 14% the physical activity guideline [8]. Whereas most parents reported declines in physical activity from pre-pandemic to Spring 2020, parents reported that children and youth’s movement behaviours in Fall 2020 were more similar to pre-pandemic levels [8]. When the results of this same study were analyzed by region in Canada, it was observed that children and youth in Atlantic Canada, the region with the least public health restrictions in Fall 2020, were more likely to meet the physical activity guidelines than children and youth living in Quebec, the region with the highest prevalence of COVID-19 cases [10]. Children and youth in Atlantic Canada also experienced smaller declines in outdoor physical activity compared to children and youth living in Quebec [10]. A study from Nova Scotia conducted in summer 2020, a time with limited physical activity and public health restrictions that limited physical activity opportunities, reported that 44% of children and youth were doing less, 40% were doing more, and 16% were doing about the same amount of physical activity as before the pandemic [4]. Our study did not assess changes in physical activity or sleep from pre-pandemic levels; however, the participants in our study did not experience changes in physical activity during Fall 2020. This may be attributed to their participation in BOKS programming during a time when Nova Scotia was experiencing its second wave of the pandemic and opportunities for organized sport and recreation outside of school were extremely limited [11]. 

Increased physical activity, as assessed with objective measures, was reported as a positive outcome of BOKS programming in before-school programming; however, we did not observe a change in parent-proxy reports of students’ physical activity [15]. Our measure of physical activity in this study asked about a child’s weekly habitual activity and was not a direct measure of physical activity within the BOKS programming. Previous research also suggests that 12 weeks of BOKS programming in a before-school program was associated with significant improvements in positive affect and a positive trend for improvements in life satisfaction and peer relationships [17]. While we did not observe changes in these three outcomes, there was a positive trend for improvements in peer relationships. It is reassuring we did not see negative changes in social-emotional outcomes, given our study was conducted during the second wave of the COVID-19 pandemic, as other work reported that children and youth’s mental health and wellbeing suffered during the pandemic. A report from UNICEF Canada reported that 69% of survey respondents said the pandemic had a negative or very negative impact on their mental health and most were concerned about maintaining peer and family relationships [1]. In Nova Scotia, parents reported their children were feeling bored and that they missed important life events and interacting with peers during the pandemic [4]. 

Our results support the idea that physical activity participation may be protective against declines in wellbeing and mental health associated with the COVID-19 pandemic. Previous work suggests that the psychological impact of the COVID-19 pandemic depends on personality traits and that certain traits such as resilience are associated with depression, anxiety, and stress [25]. The psychological stress of lockdowns was also associated with emotional eating [26]. Among 1000 children and youth in the United States, engaging in regular physical activity was associated with fewer externalizing problems, lower total difficulties, and fewer internalizing symptoms [27]. Given the psychological and emotional stress associated with the pandemic, physical activity and play should be promoted, particularly during times of severe public health restrictions, to improve children and youth’s physical, mental, and social-emotional health. As the COVID-19 pandemic continues in some jurisdictions, it is recommended that parents and caregivers incorporate family physical activity into their children’s new routines, particularly outdoors, that teachers and educators implement physically active lessons (in class with physical distancing or virtually), and that governments promote physical activity and play as part of response strategies and provide education on how it can be conducted safely, such as outdoors or outdoors with appropriate physical distancing [28]. 

This study was conducted as an evaluation of BOKS programming implemented in an existing after-school program. As a result, this study was not conducted with the rigour associated with an intervention or implementation studies, such as randomizing sites or blinding assessors. The strengths of this study include the use of a comprehensive suite of previously validated assessments for children’s health and health behaviours. The timing of our study, while not intentional, allowed us to capture the impact of BOKS programming at a time when children and youth’s opportunities for sport and recreation were limited due to public health restrictions. Due to these restrictions, our research team was not able to visit sites in person and quickly adapted the evaluation to be entirely virtual. Some limitations of this study include the small sample size and that the baseline and follow-up samples are not matched, both of which are consequences of no in-person visits to study sites. In addition, our measure of physical activity asked parents about habitual physical activity rather than physical activity levels during BOKS programming and should be addressed in future studies about the impact of BOKS programming. 

## 5. Conclusions

The COVID-19 pandemic disrupted the routines of children and youth, and opportunities for physical activity, recreation, and sport were severely limited at times of strong public health measures. We observed that the social, emotional, and cognitive health and health behaviours of children and youth who participated in BOKS programming were protected against declines associated with the COVID-19 pandemic in Nova Scotia. Our results suggest that increased physical activity opportunities or programming in school or childcare settings may provide a protective effect and mitigate some of the negative consequences of public health restrictions. Extra efforts should be made to keep children and youth active in the settings they regularly spend time in, such as school and childcare. Educators and program leaders should be provided with resources and professional development training regarding the incorporation of more movement and play in these settings. 

## Figures and Tables

**Table 1:** Participant Characteristics.

	Baseline (n = 159)	Follow-Up (n = 79)
Grade		
4	96 (60.4%)	51 (64.6%)
5	49 (30.8%)	24 (30.4%)
6	10 (6.3%)	4 (5.1%)
Other	4 (2.5%)	0
Gender		
Male (including transgender male)	75 (47.2%)	34 (43.0%)
Female (including transgender female)	83(52.2%)	43 (54.4%)
Other	1 (0.6%)	2 (2.5%)

**Table 2:** NIH PROMIS Scores.

	Baseline	Follow-Up		
	(n = 159)	(n = 79)	*t*-Value	*p*-Value
Raw scores				
Life Satisfaction	33.90 ± 4.39	34.58 ± 4.09	−1.05	0.296
Family Relationships	27.25 ± 3.65	27.08 ± 4.00	0.31	0.761
Cognitive Function	26.20 ± 5.85	27.17 ± 5.56	−1.16	0.246
Peer Relationships	28.88 ± 4.70	30.14 ± 4.29	−1.86	0.059
Positive Affect	31.21 ± 3.65	31.08 ± 3.51	0.25	0.806
Global Health	28.58 ± 3.36	28.78 ± 3.27	−0.43	0.666
Physical Activity	24.33 ± 5.89	23.64 ± 6.04	0.787	0.732
Sleep	8.21 ± 3.44	7.35 ± 3.32	1.72	0.087
Psychological Stress	8.66 ± 2.91	7.85 ± 2.86	1.896	0.059
Adjusted T-Scores				
Life Satisfaction	47.79 ± 7.43	48.26 ± 7.41	−1.08	0.332
Family Relationships	49.55 ± 7.70	49.41 ± 8.23	0.12	0.902
Cognitive Function	46.67 ± 7.15	47.80 ± 7.05	−1.08	0.281
Peer Relationships	47.56 ± 8.40	50.00 ± 8.56	−1.95	0.052
Positive Affect	47.92 ± 6.84	47.69 ± 6.66	0.23	0.817
Global Health	49.41 ± 7.51	50.04 ± 7.13	−0.61	0.544
Physical Activity	50.12 ± 6.49	49.45 ± 6.40	0.79	0.432
Sleep	55.57 ± 9.12	53.17 ± 9.20	1.78	0.076
Psychological Stress	56.37 ± 8.08	54.36 ± 8.09	1.69	0.092

Values presented as Mean ± SD. NIH: National Institute of Health; PROMIS: Patient-Reported Outcomes Measures Information System.

## Data Availability

The dataset supporting the conclusion of this article is available from the authors upon reasonable request and the completion of a data transfer agreement.
